# Association of Functional Polymorphisms of KIR3DL1/DS1 With Behçet's Disease

**DOI:** 10.3389/fimmu.2019.02755

**Published:** 2019-11-29

**Authors:** Ángel Castaño-Núñez, Marco-Antonio Montes-Cano, José-Raúl García-Lozano, Norberto Ortego-Centeno, Francisco-José García-Hernández, Gerard Espinosa, Genaro Graña-Gil, Juan Sánchez-Bursón, María-Rosa Juliá, Roser Solans, Ricardo Blanco, Ana-Celia Barnosi-Marín, Ricardo Gómez de la Torre, Patricia Fanlo, Mónica Rodríguez-Carballeira, Luis Rodríguez-Rodríguez, Teresa Camps, Santos Castañeda, Juan-Jose Alegre-Sancho, Javier Martín, María-Francisca González-Escribano

**Affiliations:** ^1^Department of Immunology, Hospital Universitario Virgen del Rocío, Instituto de Biomedicina de Sevilla (IBiS), CSIC, Universidad de Sevilla, Seville, Spain; ^2^Department of Internal Medicine, Hospital Clínico San Cecilio, Granada, Spain; ^3^Department of Internal Medicine, Hospital Universitario Virgen del Rocío, Seville, Spain; ^4^Department Autoimmune Diseases, Hospital Universitari Clínic, Barcelona, Spain; ^5^Department of Rheumatology, Complejo Hospitalario Universitario A Coruña, A Coruña, Spain; ^6^Department of Rheumatology, Hospital Universitario de Valme, Seville, Spain; ^7^Department of Immunology, Hospital Universitari Son Espases, Palma de Mallorca, Spain; ^8^Department of Internal Medicine, Autoimmune Systemic Diseases Unit, Hospital Vall d'Hebron, Universidad Autonoma de Barcelona, Barcelona, Spain; ^9^Department of Rheumatology, Hospital Universitario Marqués de Valdecilla, Santander, Spain; ^10^Department of Internal Medicine, Complejo Hospitalario Torrecárdenas, Almería, Spain; ^11^Department of Internal Medicine, Hospital Universitario Central de Asturias, Asturias, Spain; ^12^Department of Internal Medicine, Hospital Virgen del Camino, Pamplona, Spain; ^13^Deparment of Internal Medicine, Hospital Universitari Mútua Terrassa, Terrassa, Spain; ^14^Department of Rheumatology, Hospital Clínico San Carlos, Madrid, Spain; ^15^Department of Internal Medicine, Hospital Regional Universitario de Málaga, Málaga, Spain; ^16^Department of Rheumatology, Hospital de la Princesa, IIS-Princesa, Madrid, Spain; ^17^Department of Rheumatology, Hospital Universitario Doctor Peset, Valencia, Spain; ^18^Instituto de Parasitología y Biomedicina “López-Neyra”, CSIC, PTS Granada, Granada, Spain

**Keywords:** Behçet's disease, HLA, KIR, NK cells, functional polymorphisms

## Abstract

Behçet's disease (BD) is an immune-mediated vasculitis related to imbalances between the innate and adaptive immune response. Infectious agents or environmental factors may trigger the disease in genetically predisposed individuals. HLA-B51 is the genetic factor stronger associated with the disease, although the bases of this association remain elusive. NK cells have also been implicated in the etiopathogenesis of BD. A family of NK receptors, Killer-cell Immunoglobulin-like Receptor (KIR), with a very complex organization, is very important in the education and control of the NK cells by the union to their ligands, most of them, HLA class I molecules. This study aimed to investigate the contribution of certain KIR functional polymorphisms to the susceptibility to BD. A total of 466 BD patients and 444 healthy individuals were genotyped in HLA class I (A, B, and C). The set of KIR genes and the functional variants of KIR3DL1/DS1 and KIR2DS4 were also determined. Frequency of KIR3DL1^*^004 was lower in patients than in controls (0.15 vs. 0.20, *P* = 0.005, Pc = 0.015; OR = 0.70; 95% CI 0.54–0.90) in both B51 positive and negative individuals. KIR3DL1^*^004, which encodes a misfolded protein, is included in a common telomeric haplotype with only one functional KIR gene, KIR3DL2. Both, KIR3DL1 and KIR3DL2 sense pathogen-associated molecular patterns but they have different capacities to eliminate them. The education of the NK cells depending on the HLA, the balance of KIR3DL1/KIR3DL2 licensed NK cells and the different capacities of these receptors to eliminate pathogens could be involved in the etiopathogenesis of BD.

## Introduction

Behçet's disease (BD) [OMIM #109650] is a rare, chronic and systemic vasculitis characterized by recurrent oral and genital ulcers, although other clinical manifestations, such as skin lesions, ocular, gastrointestinal, and neurological disorders are relatively common. This chronic disorder is an immune-mediated disease in which imbalances between the innate and adaptive immune response triggered by infectious agents or environmental factors in genetically predisposed individuals have been suggested as the underlying mechanisms of the disease (1). Among the genetic factors, HLA-B51 has been associated with the disease in many populations (2); more recently, other genes related to the immune system such as IL23R, IL10, STAT4, CCR1, CCR3, KLRC4, ERAP1, TNFAIP3, and FUT2 have been associated with this disease (3).

Natural killer (NK) cells are lymphocytes of the innate immune system with several activators and inhibitor receptors on their surface. Among them, the Killer-cell Immunoglobulin-like Receptor (KIR) gene family consisted of a set of 15 genes: KIR2DL1, KIR2DL2, KIR2DL3, KIR2DL4, KIR2DL5A, KIR2DL5B, KIR2DS1, KIR2DS2, KIR2DS3, KIR2DS4, KIR2DS5, KIR3DL1, KIR3DL2, KIR3DL3 and KIR3DS1 and 2 pseudogenes: KIR2DP1 and KIR3DP1 located near to the leukocyte receptor complex in the chromosome 19. The KIR gene content is variable among individuals being a major contributor to the KIR diversity in the human population and the NK cell repertoires (4). At present, 625 genotypes (different sets of KIR genes) named by numbers assigned sequentially have been found in the human population^1^. Based on their gene content, two kinds of KIR haplotype groups, A and B (that give rise to the genotypes AA and Bx), have been described. Although there is no single specific criterion to distinguish them, from a practical point of view, one individual is considered as Bx when its set of KIR genes included at least one of the following genes: 2DL2, 2DL5, 3DS1, 2DS1, 2DS2, 2DS3, 2DS5, and it is assigned as AA when all these genes are absent. The KIR genes encoded activator or inhibitor molecules, in general, the molecules with long cytoplasmic tails (L) contains two immune tyrosine-based inhibitory motifs (ITIM) which transduce inhibitory signals to the NK cell, whereas the molecules with short cytoplasmic tails (S) possess a positively charged amino acid residue in their transmembrane region which allows them to associate with a DAP12 molecule generating an activation signal (4). The A Haplotypes have fewer activator genes than haplotypes B, in fact, the only two activator genes present in AA individuals are KIR2DS4 and KIR2DL4, but only KIR2DS4 binds HLA classical molecules. Besides, each KIR gene has a variable number of alleles, some of them with functional significance. Indeed, a different expression in the cell membrane of diverse KIR3DL1 alleles, ranging from practically null (in KIR3DL1^*^004, which encodes a misfolded receptor mostly retained inside the cell) to high expression, or production of soluble forms in the case of KIR2DS4 (5, 6).

The KIR-ligands are HLA class I molecules and the interaction between the pair ligand-receptor is fundamental for the regulation of NK cell activity. All the classical HLA-class I molecules (HLA-A, B and C) present peptides to the CD8+ T cells, however, not all of them are ligands of the KIR molecules. In this sense, all the HLA-C molecules are ligands of KIR and they can be grouped as C1 (Asn80) or C2 (Lys80) which are ligands of KIR2DL2 and KIR2DL1, respectively. Nevertheless, only some HLA-A and B molecules are KIR ligands, specifically, those bearing the Bw4 epitope are KIR3DL1 ligands and A3 and A11 have been described to be KIR3DL2 ligands (4). HLA-B51 and other HLA molecules associated with BD have the Bw4 epitope and interactions between Bw4 and KIR3DL1 have been proposed as one of the possible underlying mechanisms to explain the relationship between HLA class I and BD (7). However, a large number of common HLA-B alleles encoding molecules with the Bw4 epitope (e.g., B^*^44, B^*^49, etc.) have never been associated with this disease, even those with isoleucine at position 80 (Bw4-80I), which are the strongly interacting ligands. The Bw4 epitope spans the residues 77–83 but it has been described that changes at specific positions in the HLA-B molecule outside this epitope, in particular, position 97, affect the interaction of Bw4 with KIR3DL1 and, interestingly, the position 97 of HLA-B has been found strongly associated with susceptibility to BD (8–10).

With this picture, great complexity and a relative lack of the knowledge of the polymorphism of the KIR system and its relationship with its ligands, the objective of this study was to investigate the contribution of certain KIR functional polymorphisms to the susceptibility to BD.

## Materials and Methods

### Study Population

The study included a total of 466 BD-unrelated patients (44.2% males) who fulfilled the 1990 International Study Group classification criteria for BD (11), and 444 unrelated healthy individuals (50% males) included as the control group. All the subjects were Spanish European recruited from 17 Spanish hospitals across the country. The study was approved by all local ethical committees of the corresponding hospitals, and all the study participants gave their written informed consent to participate. Clinical features of the patient group were the following: 100% had oral ulcers, 59.4% genital ulcers, 53.9% uveitis, 42% arthritis, and 21% vascular, 18.2% neurological, 16.3% positive pathergy test, and 15.4% gastrointestinal involvement.

### DNA Extraction

Peripheral blood collected in EDTA tubes was obtained from the healthy controls, and peripheral blood or saliva served as starting material from patients. Genomic DNA was extracted using the QIAamp DNA Mini Kit (Qiagen, Barcelona, Spain) according to the manufacturer's recommendations and stored at −20°C. The purity of DNA was determined using a NanoDrop spectrophotometer (Thermo Fisher Scientific, Wilmington, DE, USA). Only DNA samples having a 260/280 absorbance ratio of 1.7–2.0 and a final concentration of 10–20 ng/μl were considered appropriate. A total of 14 DNA samples from saliva were eliminated because they did not meet these quality criteria.

### Genotyping

The HLA class I (A, B, and C) genotyping was carried out using a PCR-SSOP Luminex method, LABType SSO (One Lambda Inc., Canoga Park, CA), according to the manufacturer's instructions. Briefly, target DNAs were PCR-amplified using group-specific primers (HLA-A, -B, or -C) and the biotinylated-PCR products were denatured and hybridized with specific probes bound to colored-coded microspheres. Phycoerythrin conjugated to Streptoavidin was used to label and reveal reactions which were read in a flow analyzer, LABScanTM100, to identify fluorescent intensity on each microsphere. The software HLA fusion 2.0 (One Lambda Inc.) was used to assign the HLA typing of each locus. This method allows a medium resolution genotyping of each class I HLA gene. After class I genotyping, the samples were classified as A3/A11, Bw4, Bw4-80I, C1, and C2.

The set of KIR genes presents in each sample was determined also using a PCR-SSOP Luminex method, KIR SSO Genotyping Test (One Lambda Inc., Canoga Park, CA), according to the manufacturer's instructions. The principle of the method is the same as that employed for HLA-class I genotyping with the corresponding group-specific primers and bound-probes. Also, to distinguish the presence/absence of KIR genes, this method allows discriminating between some groups of alleles, specifically, between the group of KIR2DS4 alleles with the full sequence (KIR2DS4^*^001/011/014/015) which encode functional proteins and the group with a deletion of 22 base pairs (KIR2DS4^*^003/004/006/007/008/009/010/012/013) which encode non-functional variants (6).

KIR3DL1/S1 individuals were genotyped in the rs149123986 by real-time PCR using TaqMan SNP Genotyping Assays (Applied Biosystems, Barcelona, Spain) in a LightCycler 480 (Roche, Barcelona, Spain). This single nucleotide polymorphism (SNP, A/G) allows to distinguish two groups of KIR3DL1 alleles with different expression patterns: the KIR3DL1^*^004 (rs149123986G) with a very low or null membrane expression (3DL1Null) and the rest of alleles (rs149123986A), which have a normal although variable membrane expression (3DL1Exp)^2^.

### Statistical Analysis

Phenotypic and genotypic frequencies were estimated by direct counting and distributions were compared using the χ^2^ test. This test was applied to check the association of (a) different ligands: A3/11, B51, Bw4, Bw4-80I, C1, and C2; (b) the KIR genes, haplotypes and genotypes; (c) the KIR3DL1/DS1 variants in the full group and the subgroups B51 Bw4, Bw4-80I; and (d) the KIR2DS4 variants in the total groups and among individuals AA and Bx and stratified according to the presence/absence of the combination ligand and receptor. The *P*-values were corrected by Bonferroni's adjustment (Pc) considering the number of tests in each case. Those comparisons with Pc-values <0.05 were considered associated whereas those with *P* < 0.05 but with Pc > 0.05 were considered suggestive of association. The odds ratios (ORs) and 95% confidence intervals (95% CI) were calculated using the web software OpenEpi (Open Source Epidemiologic Statistics for Public Health, Versión 3.01. www.OpenEpi.com).

## Results

### HLA Molecules KIR-Ligands

A total of 435 BD patients (96.2%) and 439 controls (98.9%) were fulfilled genotyped and included in the statistical analysis. The distribution of different HLA molecules KIR-ligands is displayed in Table 1. The strongest associations were: HLA-B51, conferring risk and A3/11, conferring protection. The frequency of A3/11 was decreased in both patient groups, B51 positive and negative (18 and 21%, respectively, vs. 26 and 33% in their corresponding controls), although statistical significance was reached only in the negative group. Bw4 and Bw4-80I confer risk but with a lower OR than B51. Among the group B51 negative (250 patients and 370 controls), the distribution of Bw4 and Bw4-80I was not significantly different in patients and controls (Bw4 76.8% in patients vs. 76.2 in controls; Bw4-80I 56.0% in patients vs. 56.2% in controls, *P* > 0.05 in both cases). No differences in the distribution of C1 and C2 were detected in any case.

**Table 1:** Distribution of HLA molecules KIR-ligands in Spanish BD patients and controls.

**HLA**	**BD (%)**	**Controls (%)**	***P***	**OR (95% CI)**
	***N* = 435**	***N* = 439**		
B51	185 (42.5)	69 (15.7)	<10^−7^	3.96 (2.89–5.48)
A3/11^a^	87 (20.0)	140 (31.9)	<10^−4^	0.53 (0.39–0.73)
Bw4	377 (86.7)	351 (80.0)	0.008	1.63 (1.13–2.35)
Bw4-I80	325 (74.7)	277 (63.1)	0.0001	1.73 (1.29–2.31)
C1	329 (75.6)	351 (79.9)	>0.05	
C2	313 (71.9)	302 (68.8)	>0.05	
C1C1	122 (28.0)	137 (31.2)	>0.05	
C1C2	207 (47.6)	214 (48.7)	>0.05	
C2C2	106 (24.4)	88 (20.0)	>0.05	

a *B51 positive group: 34 patients and 18 controls A3/11 positive (p = 0.09); B51 negative group: 53 patients and 122 controls A3/11 positive (p = 0.001)*.

### KIR Genes

The distribution of the KIR genes in our cohort of patients and controls is summarized in Table 2. All the KIR genes and pseudogenes were detected in this study and four of them, corresponding to the centromeric (3DL3 and 3DP1) and telomeric (2DL4 and 3DL2) framework genes, were found in all the samples. The distribution of genes was similar in patients and controls and statistically significant differences were not reached in any case. Regarding the distribution of the KIR haplotypes, Bx was the most common in both groups and no significant differences between patients and controls were observed. Concerning the KIR genotypes, a total of 51 genotypes were found in our cohort, but only 12 had a frequency upper 2% in at least one of the groups. The genotype 2 was slightly less frequently represented in patients than in controls (8.7 vs. 14.1%, respectively, *P* = 0.01, Pc > 0.05; OR 0.58; 95% CI 0.38–0.89).

**Table 2:** Distribution of KIR genes in Spanish BD patients and healthy controls.

***KIR***	**BD**	**Controls**	***P***	**Pc**
	***n* = 435 (%)**	***n* = 439 (%)**		
**Genes**				
*2DL1*	416 (95.6)	422 (96.1)		
*2DL2*	270 (62.1)	267 (60.8)		
*2DL3*	373 (85.7)	382 (87.0)		
*2DL4*	435 (100)	439 (100)		
*2DL5A/2DL5B*	241 (55.4)	261 (59.4)		
*2DP1*	416 (95.6)	423 (96.4)		
*2DS1*	174 (40.0)	194 (44.2)		
*2DS2*	269 (61.8)	262 (59.7)		
*2DS3*	156 (35.9)	153 (34.8)		
*2DS4*	408 (93.8)	421 (95.9)		
*2DS5*	141 (32.4)	168 (38.3)		
*3DL1*	409 (94.0)	422 (96.1)		
*3DS1*	181 (41.6)	192 (43.7)		
*3DL2*	435 (100)	439 (100)		
*3DL3*	435 (100)	439 (100)		
*3DP1*	435 (100)	439 (100)		
**Haplotypes**	***n*** **=** **435 (%)**	***n*** **=** **439 (%)**		
AA	109 (25.1)	100 (22.8)		
Bx	326 (74.9)	339 (77.2)		
**Genotypes**	***n*** **=** **435 (%)**	***n*** **=** **439 (%)**		
1	109 (25.1)	100 (22.8)		
2	38 (8.7)	62 (14.1)	0.01	>0.05
3	23 (5.3)	21 (4.8)		
4	65 (14.9)	63 (14.3)		
5	39 (9.0)	45 (10.2)		
6	26 (6.0)	36 (8.2)		
7	18 (4.1)	16 (3.6)		
13	10 (2.3)	4 (0.9)		
70	9 (2.1)	5 (1.1)		
71	8 (1.84)	12 (2.7)		
72	14 (3.2)	11 (2.5)		
73	12 (2.8)	10 (2.3)		
The rest^a^	64 (14.7)	54 (12.3)		

a *Genotypes with a frequency lower than 2%*.

### Functional KIR Polymorphyms

Data regarding the polymorphism of KIR3DL1/S1 are displayed in Table 3. The distribution of individuals KIR3DL1/S1 was not significantly different in patients and controls (KIR3DL1+DS1+ 35.6 vs. 39.9%, KIR3DL1+DS1– 58.3 vs. 56.3%, KIR3DL1-DS1+ 6.0 vs. 3.9%, respectively). Nevertheless, the distribution of the genotypes of rs149123986 was significantly different in both groups (*p* = 0.01 in 2 × 3 contigency Table) with an over-representation of AA in patients (73.5% vs. 64.3 in controls, Pc = 0.006; OR = 1.54; 95% CI 1.16–2.07) and a down-representation of AG (23.0 vs. 31.7%, Pc = 0.015, OR = 0.65; 95% CI 0.48–0.88). The distribution of the individuals in the six possible groups taking into account both, the KIR3DL1/S1 and the rs149123986, was different in patients and controls (*p* = 0.002 in 2 × 6 Table) with down-representation of 3DL1Null/3DL1 (6.2 vs. 11.4%, *p* = 0.007, Pc = 0.042; OR = 0.51; 95% CI 0.32–0.84) and a trend to an over-representation of 3DL1Exp/3DL1Exp (38.1 vs. 31.9%, *p* = 0.052) individuals among patients. The distribution of the three allele groups: 3DL1Exp, 3DL1Nul, and 3DS1 was different in patients and control (*p* = 0.01, in 2 × 3 contigency Table) having the group of patients a decreased frequency of 3DL1Null (0.15 vs. 0.20, *P* = 0.005, Pc = 0.015; OR = 0.70; 95% CI 0.54–0.90) and a slight increased frequency of 3DL1Exp (0.61 vs. 0.56, *P* = 0.03, Pc > 0.05). Results were similar among B51 positive and negative individuals with a suggestive down-representation of 3DL1Null/3DLS1 in both groups (B51positive: 5.9% in patients vs. 15.9% in controls *P* = 0.009 Pc > 0.05; OR = 0.34; 95% CI 0.13–0.83 and B51 negative: 6.4% in patients vs. 10.5% in controls *P* = 0.04 Pc > 0.05; OR = 0.58; 95% CI 0.3–1.05) (Table 4).

**Table 3:** Frequency of the KIR3DL1/DS1 functional polymorphism in BD patients and controls.

**KIR**		**rs149123986**		**BD**		**Controls**		***P***	**Pc**	**OR (95%CI)**
***3DL1***	***3DS1***			***n* = 435**	**%**	***n* = 439**	**%**			
+	–	AA	*3DL1^*Exp*^/3DL1^*Exp*^*	166	38.1	140	31.9	0.05	>0.05	
+	–	AG	*3DL1^*Exp*^/3DL1^*Null*^*	74	17.0	89	20.3			
+	+	AA	*3DL1^*Exp*^/3DS1*	128	29.4	125	28.5			
+	–	GG	*3DL1^*Null*^/3DL1^*Null*^*	14	3.2	18	4.1			
**+**	**+**	**AG**	***3DL1**^***Null***^**/3DS1***	**27**	**6.2**	**50**	**11.4**	**0.007**	**0.042**	**0.51 (0.32–0.84)**
**-**	+	AA	*3DS1/3DS1*	26	6.0	17	3.9			
					AF		AF			
			*3DL1^*Exp*^*	534	0.61	494	0.56	0.03	>0.05	
			***3DL1**^***Null***^*	**129**	**0.15**	**175**	**0.20**	**0.005**	**0.015**	**0.7 (0.54–0.90)**
			*3DS1*	207	0.24	209	0.24	>0.05	>0.05	

*AF, allelic frequency. Bold values are those statistically significant after correction*.

**Table 4:** Frequency of the *KIR3DL1* functional polymorphism in BD patients and controls stratified according to the HLA-B51.

	**B51+**				**B51**–			
	**BD**		**Controls**		**BD**		**Controls**	
	**185**		**69**		**250**		**370**	
	***n***	**%**	***n***	**%**	***n***	**%**	***n***	**%**
*3DL1^*Exp*^/3DL1^*Exp*^*	76	41.1	25	36.2	90	36.0	115	31.1
*3DL1^*Exp*^/3DL1^*Null*^*	28	15.1	11	15.9	46	18.4	78	21.1
*3DL1^*Exp*^/3DS1*	53	28.7	19	27.5	75	30.0	106	28.6
*3DL1^*Null*^/3DL1^*Null*^*	7	3.8	3	4.3	7	2.8	15	4.1
*3DL1^*Null*^/3DS1*	11	5.9	11	15.9	16	6.4	39	10.5
*3DS1/3DS1*	10	5.4	0	0.0	16	6.4	17	4.6
*3DL1^*Exp*^*	233	0.63	80	0.58	301	0.60	414	0.56
*3DL1^*Null*^*	53	0.14	28	0.20	76	0.15	147	0.20
*3DS1*	84	0.23	30	0.22	123	0.25	179	0.24

Regarding the deletion of 22 bp of 2DS4, differences in the distribution of individuals with at least one copy of the 2DS4Full between patients and controls were not detected (42.7 vs. 37.5%, *P* = 0.13). Since 2DS4 is the only activator binding classical HLA molecules in AA individuals, patients and controls were stratified according to their haplotype group in AA or Bx but no differences were detected (Data not showed).

## Discussion

The main finding of this study is the association of the KIR3DL1^*^004 with susceptibility to BD. This allele is protective in the development of the disease and, according to our results, its effect is independent of HLA-B51.

Previous studies analyzed the relationship between the distribution of the KIR genes and the disease. Similarly to our results, no differences between patients and controls have been reported in these studies (12–15). Only one of these studies, performed in the Iranian population, investigated KIR genotypes. That study reported a different distribution of various KIR genotypes, including genotype 2, although the direction of the association was contrary to that found in the present study (15).

KIR3DL1/DS1 has focused on interest because of its function as the receptor of the Bw4 epitope. The distribution of KIR3DL1 and 3DS1 in BD was evaluated in a large cohort of Turkish. Similarly to our results, no association was found in the whole cohort neither in subgroups with B51, Bw4, or Bw4-80I (16) but this study did not investigate variants on this gene. Variants in KIR3DL1 have been reported as associated with the disease in two high throughput studies (10, 17), although none of them was designed to investigate the possible involvement of functional variants in this disease. Very recently, during the submission of this paper, a study that addresses this question in a smaller cohort composed of ethnically-mixed patients and controls was published (18). The main conclusion of that study is the same as ours, an association of the functional alleles of KIR3DL1, independent of HLA-B51 with the disease. Nevertheless, they are some discrepancies in the associated genotypes between these two studies. When the allele frequencies of the study by Petrushkin et al. are deduced of their data, results are similar to ours regarding the allele KIR3DL1^*^004, which is underrepresented in patients (with similar OR), although the statistical significance is only reached in our cohort. Nevertheless, we have opposite results concerning KIR3DL1Exp and KIR3DS1. In our cohort, the alleles that encode KIR3DL1Exp are significantly overrepresented among patients whereas, in its cohort, it is KIR3DS1 which tends to be overrepresented among patients. In our opinion, the distribution of the allelic frequencies reinforces the idea of association with the allele KIR3DL1^*^004. Discrepancies could be due to different reasons, among them, methods used in KIR3DL1 genotyping, approaches, stratification of cohorts with a relatively small number of individuals and ethnical differences. Besides, there are previous studies in other class-I pathologies such as psoriatic disease (PD) and ankylosing spondylitis (AS), in which a protective association of the KIR3DL1^*^004 allele has been reported (19–21).

KIR3DL1^*^004 encodes a misfolded receptor which is retained in the cell, although it has been described that a small quantity of folded protein can be exported to the membrane (22). One possible explanation to the protective effect of KIR3DL1^*^004 in these pathologies is that this misfolded molecule retains intracellularly the corresponding class I-risk molecules. In this case, differences in the distribution of KIR3DL1^*^004 among patients with and without the risk molecule would be expected, nevertheless, no differences were found in the present study neither in other pathologies (20, 21, 23) and no evidence supporting the intracellular interaction between KIR3DL1 and MHC class I molecules have been found (5). Interestingly, KIR3DL1^*^004 is within a common telomeric haplotype Tel-A1-V which, also, bears two alleles encoding secreted receptors, KIR2DL4^*^008 and KIR2DS4^*^006 and another that encodes a membrane receptor KIR3DL2^*^003/005/011/020 (24)^3^. Therefore, this Tel-A1-V haplotype, protective in BD, carries non-functional alleles with the only exception of KIR3DL2. Consequently, KIR3DL1^*^004 could be a marker of the Tel-A1-V haplotype and the protective association found in our study could be explained by the fact that KIR3DL2 is the only functional gene in this haplotype.

KIR3DL2 is an inhibitor framework gene, found in 100% of the population and it has many allelic forms encoding many different proteins (161 and 111, respectively, in the last database), the relative expression levels of these different proteins are unknown. The KIR3DL2 HLA-ligands are A3 and A11 and there is some evidence that KIR3DL2 can bind these HLA molecules *in vivo* (25). The education of the NK cells is based on the interaction between the inhibitory receptors and the self-MHC-I molecules, only those individuals bearing the HLA-ligand molecules have licensed NK cells that express the corresponding receptor and therefore, only the individuals A3 and A11 have mature NK cells expressing KIR3DL2. Noticeably, similarly to the findings in the present cohort, different studies in several populations have found a protective association of HLA-A3 with BD, independent of HLA-B51 (9, 10, 26). This protective effect of HLA-A3, not yet explained, could be based on the presence of licensed KIR3DL2 NK cells in these individuals.

KIR molecules having 3 extracellular domains (KIR3DL1 and KIR3DL2) are pattern recognition receptors (PRR), they sense pathogen-associated molecular patterns (PAMPs), specifically single-stranded microbial DNA molecules containing CpG motifs (CpG-ODN). KIR3DL2 binds CpG-ODN by the D0 domain and the complexes are internalized and recognized by TLR9 located in the endosomal compartment what results in cell activation. Nevertheless, in spite of KIR3DL1 binds CpG-ODN, the complexes are not internalized (27). Thus, one possible explanation to the protective effect of the Tel-A1-V haplotype is that the lack of expression of KIR3DL1 in these individuals permits a better efficiency to eliminate pathogens by KIR3DL2 NK cells. In other words, NK cells of individuals with the KIR3DL1Exp would be lesser efficient in eliminating pathogens resulting in a perpetuation of the release of inflammatory cytokines. In this sense, a protective effect of KIR3DL1^*^004 against the progression of the VIH infection to AIDS has been described (28). This mechanism would explain why the association is independent of B51 and Bw4 in our study but also psoriasis. Moreover, some HLA-Bw4 molecules, such as B27 (associated with ankylosis spondylitis) and B51 could be more efficient to license KIR3DL1 NK cells and this could be related to its association with inflammatory diseases. In this sense, the activity of ERAP1, an enzyme that has been related in an epistatic way with the HLA molecules involved in these pathologies and that can conditioner the repertoire of peptides binding to them, could modify the role of the diverse HLA-Bw4 molecules in these diseases (29).

Lastly, the Tel-A1-V haplotype includes KIR2DS4^*^006, which has the deletion of 22 base pairs, but according to our results, this deletion does not influence the disease susceptibility.

In conclusion, our results suggest a protective role of a telomeric KIR haplotype with only one functional gene in BD. Therefore, the education of the NK cells depending on the HLA, the balance of licensed KIR3DL1/KIR3DL2 NK cells and the different capacities of these receptors to eliminate pathogens could be involved in the etiopathogenesis of BD.

## Data Availability Statement

The raw data supporting the conclusions of this article will be made available by the authors, without undue reservation, to any qualified researcher.

## Ethics Statement

The studies involving human participants were reviewed and approved by CEI de los Hospitales Universitarios Vírgen Macarena-Virgen del Rocío. The patients/participants provided their written informed consent to participate in this study.

## Author Contributions

M-FG-E: experimental design. ÁC-N: experimental procedures performance. M-AM-C and J-RG-L: experimental support. ÁC-N, M-AM-C and M-FG-E: data analysis and preparation of the manuscript. NO-C, F-JG-H, GE, GG-G, JS-B, M-RJ, RS, RB, A-CB-M, RG, PF, MR-C, LR-R, TC, SC, J-JA-S, and JM: samples and clinical data providers.

### Conflict of Interest

The authors declare that the research was conducted in the absence of any commercial or financial relationships that could be construed as a potential conflict of interest.

## Footnotes

^1^http://www.allelefrequencies.net/kir6001a.asp

^2^https://www.ebi.ac.uk/ipd/kir/align.html

^3^https://www.ebi.ac.uk/ipd/kir/sequenced_haplotypes.html
